# Apigenin inhibits colonic inflammation and tumorigenesis by suppressing STAT3-NF-κB signaling

**DOI:** 10.18632/oncotarget.22145

**Published:** 2017-10-27

**Authors:** Xiao-Yu Ai, Yuan Qin, Hui-Jua Liu, Zhan-Hong Cui, Meng Li, Jia-Huan Yang, Wei-Long Zhong, Yan-Rong Liu, Shuang Chen, Tao Sun, Hong-Gang Zhou, Cheng Yang

**Affiliations:** ^1^ State Key Laboratory of Medicinal Chemical Biology and College of Pharmacy, Nankai University, Tianjin, China; ^2^ Tianjin Key Laboratory of Molecular Drug Research, Tianjin International Joint Academy of Biomedicine, Tianjin, China

**Keywords:** apigenin, IBD, CAC, NF-κB, STAT3

## Abstract

Apigenin is a naturally occurring compound with anti-inflammatory, antioxidant, and anticancer properties. Here, we investigated the effects of apigeninin inflammatory bowel disease (IBD) and colitis-associated cancer (CAC). Apigenin effectively inhibited ulcerative colitis, a type of IBD, and CAC. Apigenin decreased myeloperoxidase (MPO), inflammatory cytokine and COX-2 levels, and it attenuated inflammatory cell infiltration in treated colon tissues as compared to untreated model colon tissues. Apigenin also reduced NF-κB and STAT3 activity *in vitro* and *in vivo*, thereby inhibiting inflammation and inflammation-induced carcinogenesis. Thus apigenin appears to inhibit inflammation and inflammation-induced carcinogenesisin IBD and CAC by suppressing STAT3-NF-κB signaling.

## INTRODUCTION

Apigenin (4′,5,7-trihydroxyflavone, 5,7-dihydroxy-2-(4-hydroxyphenyl)- 4H-1-benzopyran-4-one), a naturally-occurring plant flavone present in many fruits, vegetables, and herbs, has anti-inflammatory, antioxidant, and anticancer effects [1]. The anti-inflammatory effects of apigenin have been extensively investigated [2, 3].

Inflammatory bowel disease (IBD) is a common disease affecting millions of people, and its incidence increases rapidly every year [4]. Accumulating evidence indicates that colitis-associated cancer (CAC) is strongly associated with IBD [5, 6]. In IBD, inflammatory cytokines contribute to the formation of a tumor-supportive microenvironment [7, 8]. Chronicinflammation increases the risk of CAC, which often leads to death; CAC is therefore regarded a serious complication of IBD [9, 10]. However, the efficacy of the drugs currently used to treat IBD and prevent CAC is relatively poor [11]. A better understanding of the association between inflammation and colon cancer may lead to the identification of novel methods for preventing CAC.

Activation of the transcription factor nuclear factor-kappaB (NF-κB) regulates various genes involved in early inflammatory response [12]. The NF-κB signaling pathway plays a pivotal role in the development and maintenance of intestinal inflammation. NF-κB signaling is activated in mucosal IBD cells and in colorectal carcinoma patients. Modulation of NF-κB activity is therefore a target of IBD treatments [13]. Moreover, activation of oncogenic transcription factors, such as signal transducer and activator of transcription 3 (STAT3), induces colorectal carcinoma. Greatly elevated levels of inflammatory cytokinesin early colonic lesions in a mouse model of colorectal cancer are associated with enhanced STAT3/NF-kB activation [14]. Additionally, a positive feedback loop maintains epigenetic transformations in neoplastic cells for many generations in the absence of an inducing signal [15].

We previously showed that apigenin inhibits epithelial-mesenchymal transition in hepatocellular carcinoma via the NF-κB signaling pathway [16]. Here, we examined whether apigenin exerts anti-IBD and –CAC effects by modulating the STAT3-NF-κB pathway.

## RESULTS

### Apigenin protects against chronic UC in mice

**Figure 1:**
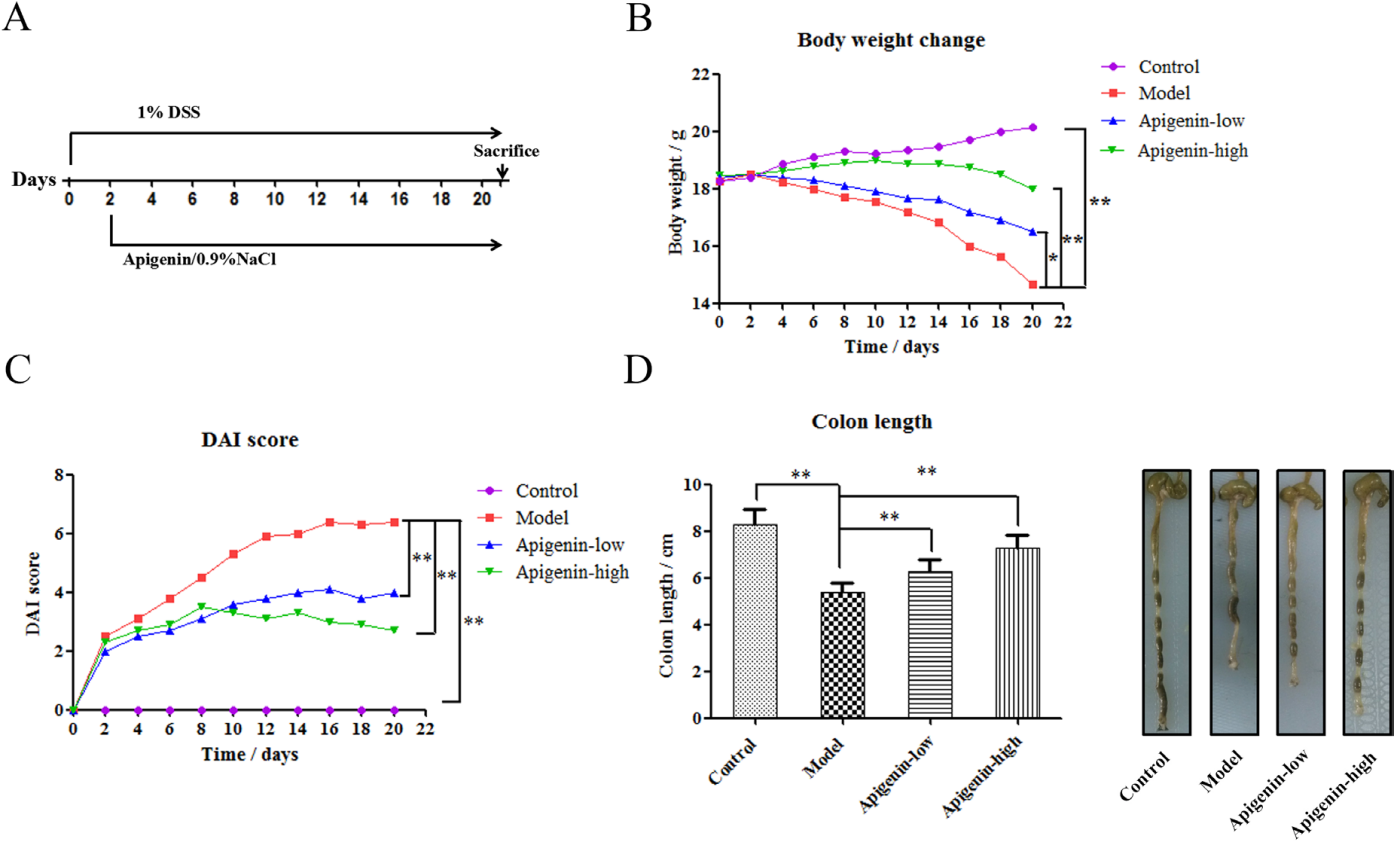
Apigenin protects against chronic UC in mice **(A)** Model of chronic DSS-induced UC in mice. **(B)** Body weights (g) of chronic UC group mice. **(C)** DAI values of chronic UC groupmice. **(D)** Average colon lengths for each group. Data are presented as means of three experiments; error bars represent standard deviation (^*^P < 0.05, ^**^P <0.01).

The effects of lentinan treatment on IBD were examinedusing chronic UC mouse models. We induced chronic UC in C57BL/6 mice through oral administration of 1% DSS for 21 days. Apigenin was administered 24 h after DSS administration (Figure 1A). DSS inhibited normal growth and reduced mouse body weights, and apigenin ameliorated weight loss in a dose-dependent manner(Figure 1B). DAI values were lower in the apigenin group than in the model group, which was consistent with the observed effects on bodyweight (Figure 1C). As shown in Figure 1D, apigenin also inhibited DSS-induced colon shortening in a dose-dependent manner.

### Apigenin inhibits colon damage and inflammatory cytokine production in UC mice

**Figure 2:**
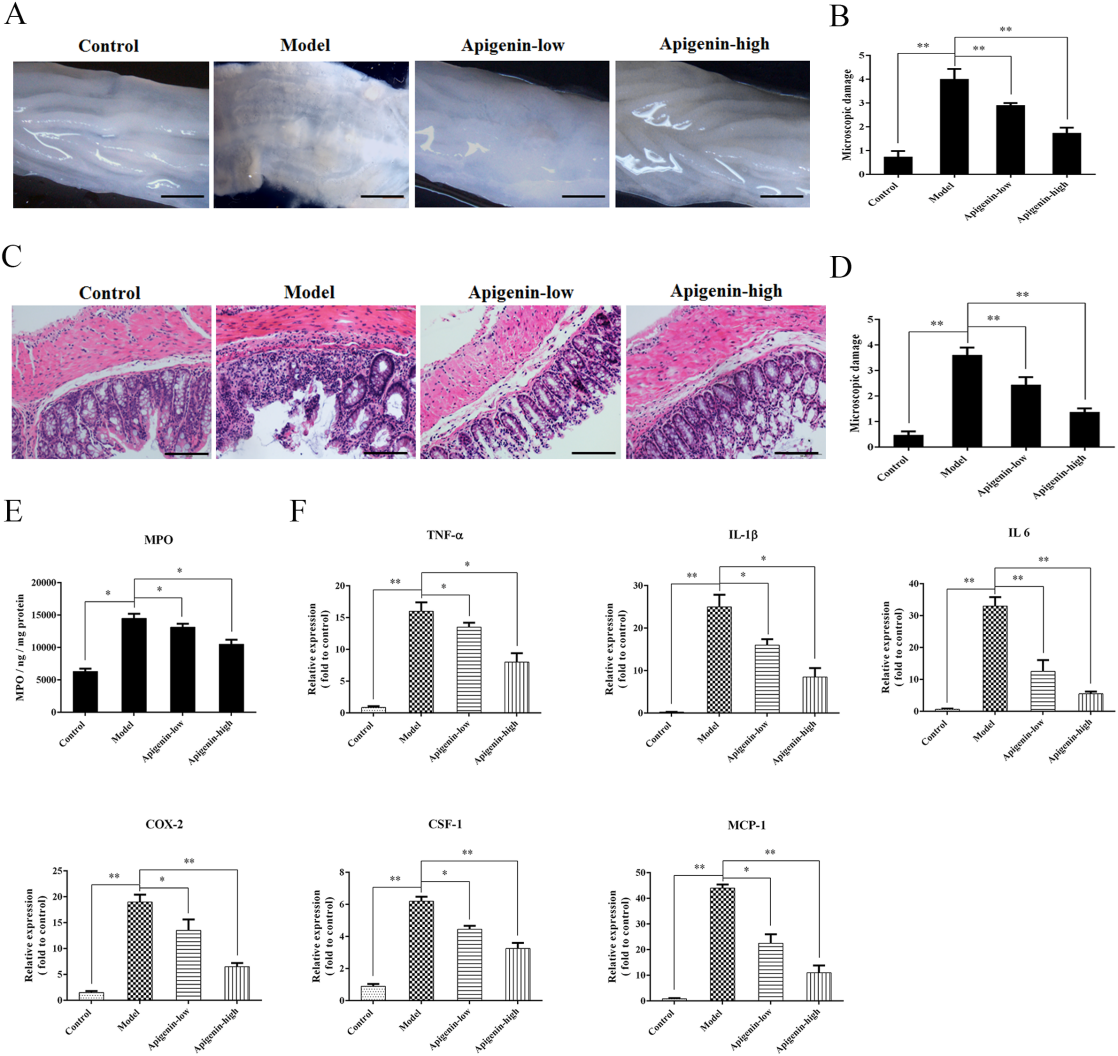
Apigenin inhibits colon damage and inflammatory cytokine production in UC mice **(A** and **B)** Photographs of colons from chronic UC mice and associated macroscopic damage scores, respectively. **(C** and **D)** Photographs of pathological sections of H&E-stained colon from chronic UC mice and associated microscopic damage scores, respectively. **(E)** MPO levels for each group. **(F)** Levels of the inflammatory cytokines TNF-α, IL-1β, IL-6, MCP-1, and CSF-1 and of COX-2. Data are presented as means of three experiments; error bars represent standard deviation (^*^P < 0.05, ^**^P <0.01).

Colon tissues fromchronic UC mice displayed granulated intestinal mucosa, superficial ulcer formation, and local mucosal hyperemia and edema. Apigenin ameliorated these types of colonic damage and reduced colon macroscopic damage scores(Figure 2A). Severe inflammatory lesions, crypt erosion, and immune cell infiltration were observed in histological sections of colorectal tissues from chronic UC mice. Apigenin reduced the severity of colitis, thereby reducing microscopic damage scores(Figure 2B).

MPO levels were higher in the UC model group than in the control and apigenin groups. Levels of the inflammatory cytokinesTNF-α, IL-1β, IL-6, MCP-1, and CSF-1 and of COX-2 were measured in mice with chronic colitis. All cytokine and COX-2 levels were higher in the model group than in the control group. Moreover, apigenin decreased all cytokine and COX-2 levels in a dose-dependent manner.

### Apigenin protects against CAC in mice

**Figure 3:**
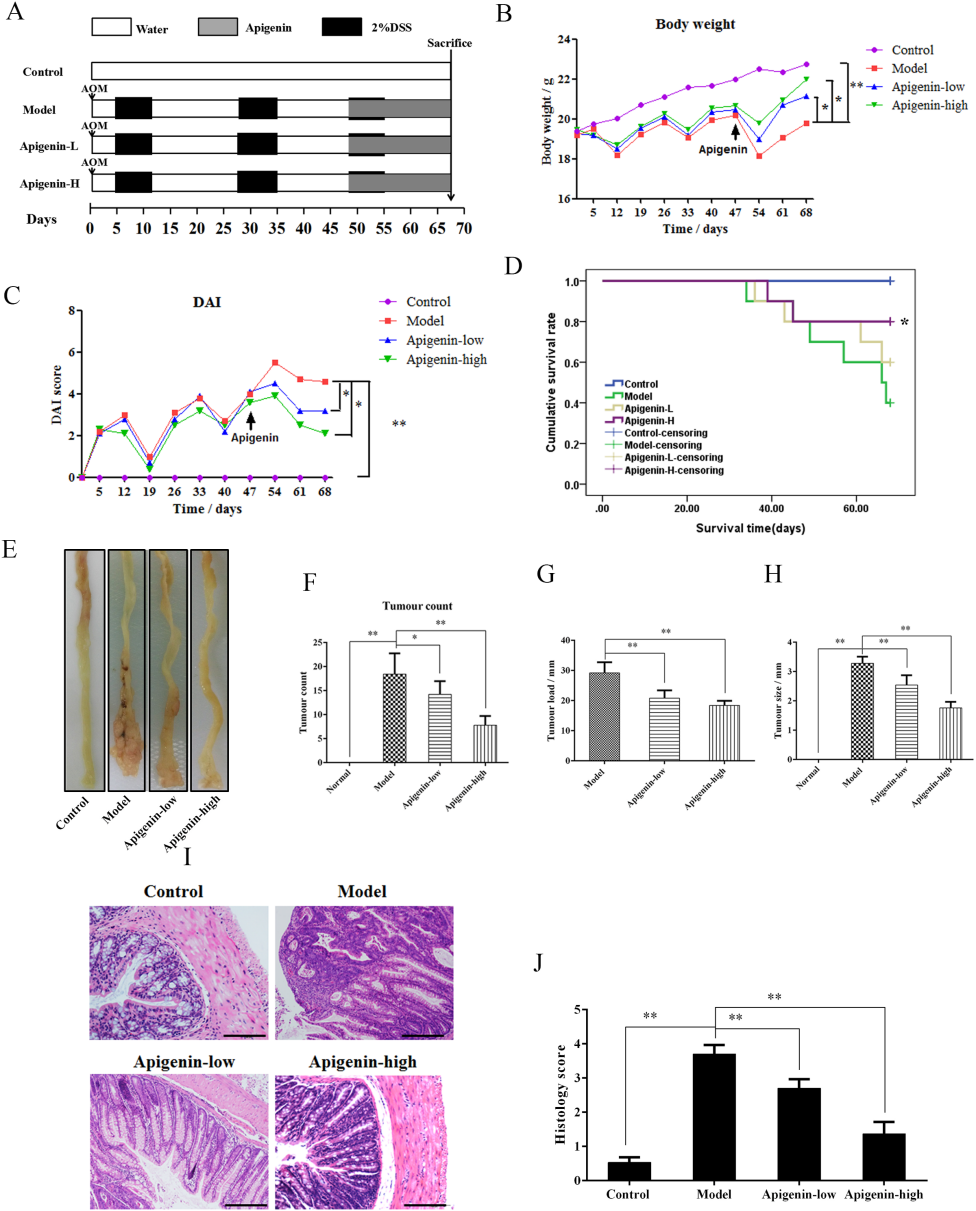
Apigenin protects against CAC in mice **(A)** Mouse model of AOM/DSS-induced CAC. **(B)** Body weights (g) of CAC mice. **(C)** DAI values of CAC mice. **(D)** Survival curves for each group. **(E)** Colon morphology of each group. **(F–H)** Tumor count, tumor load, and tumor sizesfor each group, respectively. **(I** and **J)** Photographs of pathologic colon sections from each group and associated colon histological scores, respectively. Data are presented as means of three experiments; error bars represent standard deviation (^*^P < 0.05, ^**^P <0.01).

An animal model of CAC was established to assess the protective effects of apigenin(Figure 3A). Mouse body weights decreased substantially after each round of exposure to DSS; this weight loss was partially reversed when DSS was withdrawn. Apigenin dose-dependently inhibited weight loss compared to model group mice (Figure 3B). DAI values were lower in the apigenin group than in the model group, consistent with the bodyweight results (Figure 3C). Survival curves showed that apigenin treatment increased the survival rate in CAC mice (Figure 3D). Macroscopic colon images are shown in Figure 3E. The average number of tumors per mouse, tumor load, and tumor size were all lower in apigenin-treated mice than in model group mice (Figure 3F–3H). Histological examination revealed that apigen in treatment reduced tumor numbers and sizes and attenuated inflammatory cell infiltration, atypical hyperplasia, and nuclear atypia in treated colon tissues compared to colon tissues from model group mice (Figure 3I and 3J).

### Apigenin inhibits production of inflammatory cytokines and colon cancer markers

**Figure 4:**
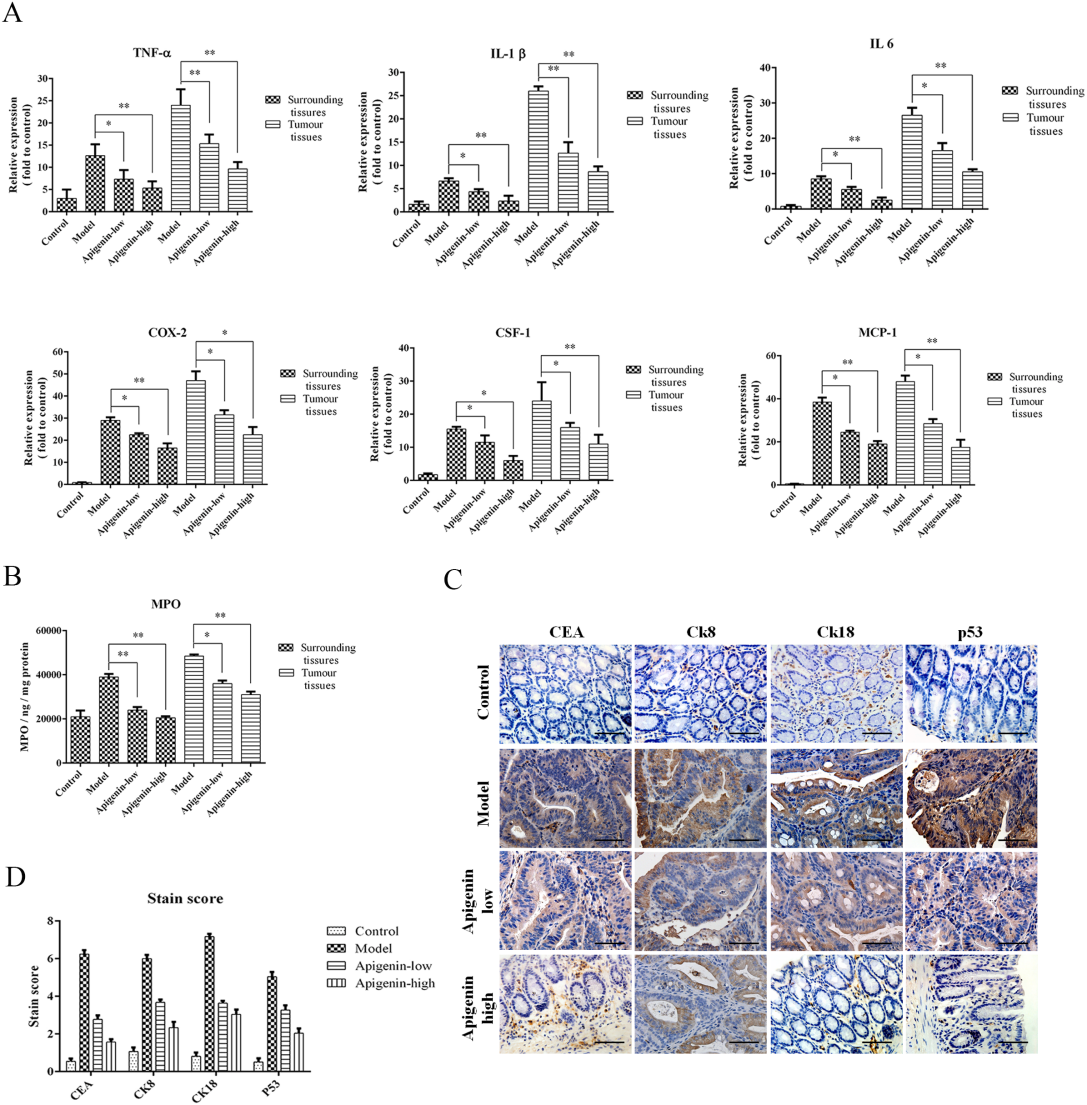
Apigenin reduces levels of inflammatory cytokines and colon cancer markers **(A)** Colon levels of the inflammatory cytokines TNF-α, IL-1β, IL-6, MCP-1, and CSF-1 and of COX-2foreach group. **(B)** MPO levels for each group. **(C)** Representative photos of IHC staining of the cancer markers CEA, CK8, CK18, and p53. **(D)** Staining scores for the cancer markers and cytokines listed above. Data are presented as means of three experiments; error bars represent standard deviation (^*^P < 0.05, ^**^P <0.01).

Levels of the inflammatory cytokines TNF-α, IL-1β, IL-6, MCP-1, and CSF-1 and of COX-2were increased in tumors andsurrounding tissues inmodel group mice compared to the other groups. Moreover, inflammatory cytokine and COX-2 levels were higher in tumor tissues than in the surrounding tissues (Figure 4A). MPO levels in tumor and surrounding tissues were also increased in the model group compared to the other groups and were higher in tumor tissues than in surrounding tissues (Figure 4B). An IHC assay showed that apigenin reduced expression of the colon cancer markers CEA, CK8, CK18, and p53in colorectal tissues from CAC mice (Figure 4C and 4D).

### Apigenin inhibits the STAT3/NF-κB pathway in colon carcinoma cells

**Figure 5:**
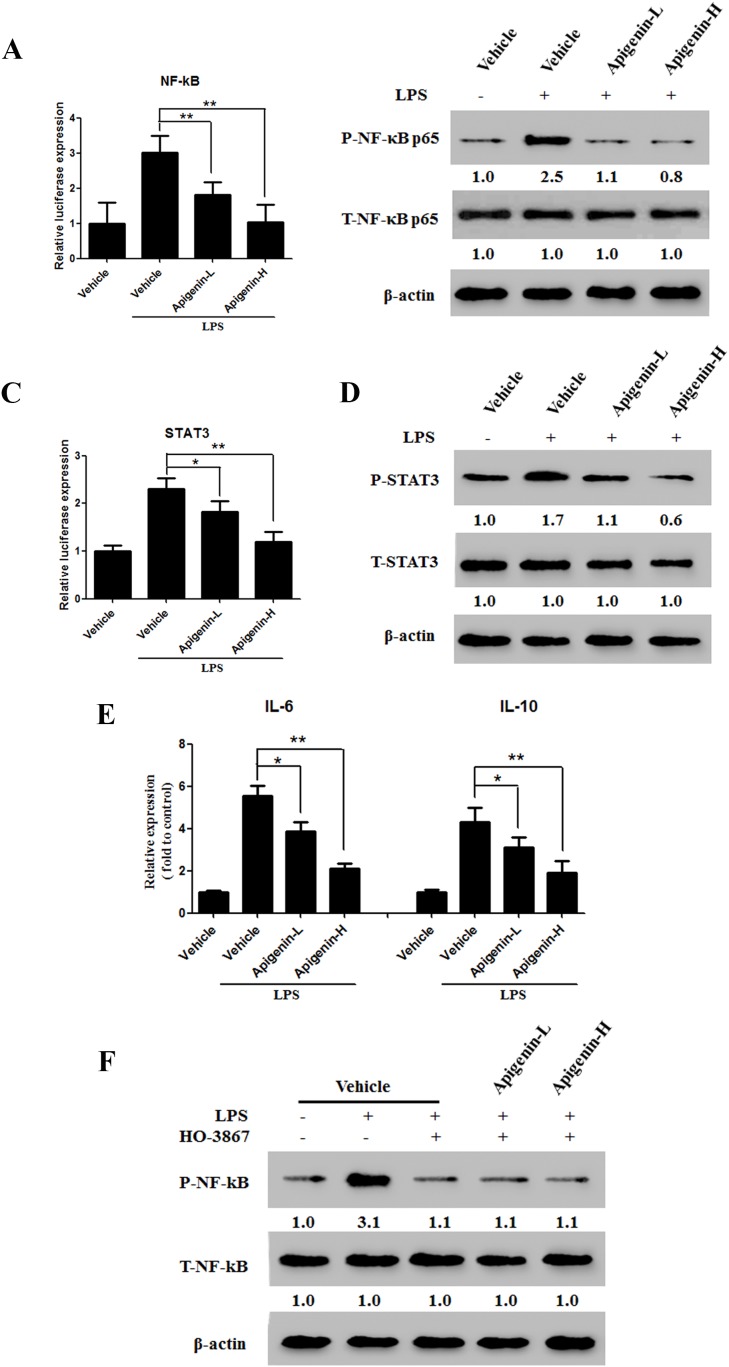
Apigenin suppresses the NF-κB/STAT3 pathway in colon carcinoma cells **(A)** Dual-luciferase assay results for NF-κB transcription in HCT-116 cells. **(B)** Levels of p-NF-κB p65 and T-NF-κB p65 after LPS administration in HCT-116 cells treated with apigenin or vehicle. **(C)** Dual-luciferase assay results for STAT3 in HCT-116 cells. **(D)** Levels of p-STAT3 and T-STAT3 after LPS administration in HCT-116 cells treated with apigenin or vehicle. **(E)** Levels of secreted IL-6 and IL-10 after LPS administration in HCT-116 cells treated with apigenin or vehicle. **(F)** Expression of p-STAT3 and T-STAT3 in HCT-116 cells pretreated with HO-3867.

Lipopolysaccharides (LPSs) are Toll-like receptors (TLRs) that activate the NF-κB signaling pathway. We used are porter gene assay and Western blot analysis to examine the effects of apigenin on NF-κB expression and activation induced by 5 μg/mL LPS in the HCT-116 colonic epithelial cancer cell line. Apigenin downregulated NF-κB in a dose-dependent manner(Figure 5A and 5B). Previous studies have demonstrated that STAT3 interacts with NF-κB in cancer cells. As shown in Figure 5C and 5D, apigenin also downregulated STAT3 in a dose-dependent manner. We then examined the effects of apigenin on LPS-induced IL-6 and IL-10 secretion. Apigenin reduced levels of both IL-6 and IL-10 (Figure 5E). Next, HCT-116 cells were treated either with or without LPS in the presence or absence of HO-3867 (20μM), a specific STAT3 inhibitor. Pretreatment with HO-3867 attenuated LPS-induced NF-κB phosphorylation (Figure 5F), but apigenin did not further reduce NF-κB phosphorylation (Figure 5F).

### Apigenin inhibits the STAT3/NF-κB pathway in colitis-associated colon cancer tumor tissues

**Figure 6:**
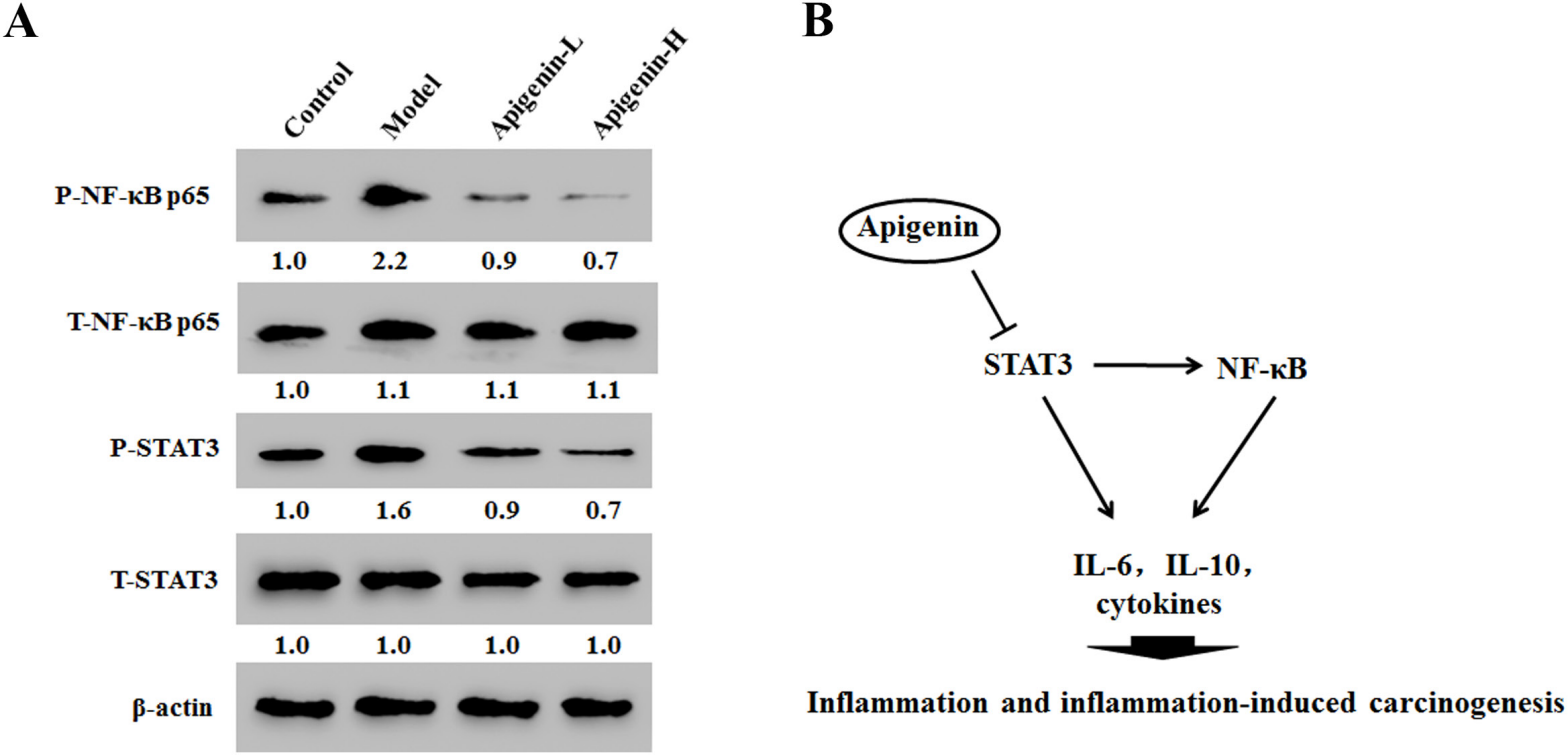
Apigenin inhibits NF-κB/STAT3 pathway protein expression in tumor tissues in colitis-associated colon cancer (CAC) tumor tissues **(A)** Results of Western blot analysis of NF-κB p65 and STAT3 expression in CAC tumor tissues. **(B)** Model showing the role of apigenin as a negative regulator of STAT3-NF-κB pathway.

In cancer tissues, apigenin reduced phosphorylation of both NF-κB at p65 and STAT3 in a dose-dependent manner (Figure 6A). These results indicate that apigenin inhibited NF-κB activation by inhibitingSTAT3 activity (Figure 6B).

## DISCUSSION

Apigenin has anti-inflammatory and anti-tumor activities in various cancer cells with little cytotoxicity, but the mechanisms underlying these effects remain unclear [17–19].

Our results showed that apigenin was an effective treatment againstchronic UC. Apigenin reduced levels of MPO, inflammatory cytokines, and COX-2 andattenuated inflammatory cell infiltration in colon tissues compared to untreatedmodel colon tissues. Long-term IBD increases the risk of CAC [20]. In IBD, inflammatory cytokines contribute to the formation of a tumor-supportive microenvironment [21].

Our results also demonstrated that apigenin inhibited CAC by reducing neutrophil infiltration and levels of colon cancer markers, MPO, inflammatory cytokines, and COX-2. Histological examination revealed that apigenin treatment reduced tumor counts and average tumorsize andattenuated inflammatory cell infiltration, atypical hyperplasia, and nuclear atypia in colon tissue compared to untreated model colon tissues. Additionally, apigenin increased mediansurvival times in mice compared to mice in the untreated model group.

We previously found that apigenin inhibits hepatocellular carcinomavia the NF-κB signaling pathway [16]. Studies suggest that NF-κB signaling is a critical link between inflammation and carcinogenesis [22].Moreover, NF-κB activation plays a key role in the upregulation of intestinal epithelial permeability [23]. LPS induces the activation of multiple proinflammatory transcription factors and signaling pathways, including NF-κB [24]. Furthermore, the NF-κB signaling pathway plays a central role in mediating inflammatory signalsand in controlling the production of proinflammatory mediators, especially in IBD and CAC [22]. TLR4 ligation by LPS promotes phosphorylation of NF-κB and degradation of I-κB, which liberates NF-κB and leads to its nuclear translocation and transcriptional activation [25]. Here, apigenin downregulated NF-κB phosphorylation in a dose-dependent manner in LPS-treated HCT-116 cells. This result differsfrom our previous findings that apigenin treatment decreased both NF-κB expression and activity in human hepatocellular carcinoma cells. The effects of apigenin on NF-κB might therefore vary in different cancer cells.

STAT family proteins, especially STAT3, are closely associated with NF-κB signaling [26–28]. Studies in cancercells demonstrated that direct interactions between STAT3 and NF-κB contribute to inflammation [29]. Moreover, this interaction is facilitated primarily by IL-6, which forms anNF-κB-IL-6-STAT3 loop that prolongs NF-κB activation in cancer cells [30]. Persistently activated STAT3 maintains constitutive NF-κB activity in tumors [29]. STAT3 and NF-κB are involved in the interplay between immune/inflammatory and malignant cells, and activation of these transcription factors promotes CRC cell proliferation and survival [30, 31]. We therefore examined whether apigenin alters activation of STAT3 pathway in this study. Apigenin downregulated STAT3 phosphorylation, but not total STAT3 levels. However, apigenin did not further attenuate NF-κB phosphorylation after pretreatment with HO-3867, a STAT3 inhibitor. This result indicates that apigenin inhibited the NF-κB-STAT3 loopby directly inhibiting STAT3, which in turn inhibitedNF-κB. Apigenin-induced inhibition ofSTAT3 and NF-κBmay therefore becrucial to itsability to inhibit colonic carcinoma progression.

In summary, our results suggest that apigenin inhibits inflammation and inflammation-induced carcinogenesis in general, and IBD and CAC specifically, by suppressing STAT3-NF-κB signaling. These findings provide anew mechanistic basis for the therapeutic application of apigenin for the treatment of inflammation-induced carcinogenesis in patients withIBD and CAC.

## MATERIALS AND METHODS

### Reagents

Apigenin was obtained from Meilunbio (Dalian, China). Dextran sulfate sodium (DSS) (MW 36,000–50,000) was purchased from MP Biomedicals (Santa Ana, California, USA). Azoxymethane (AOM) was procured from Sigma-Aldrich Co. (St. Louis, MO, USA). Sodium carboxymethylcellulose (CMC-Na) was purchased from Sangon Biotech (Shanghai, China). Lipopolysaccharide (LPS) was obtained from Sigma-Aldrich Co. (St. Louis, MO, USA). Antibodiesfor CEA, Ck8, Ck18, p53, p-NF-κB-p65, T-NF-κB-p65, p-state-3, T-state-3, and β-actin were purchased from Affinity Bioreagents (Colorado, USA). BCA assay kits were obtained from Thermo (USA). The STAT3 inhibitor HO-3867 was purchased from Selleck Chemicals (Houston, TX, USA). ELISA kits were purchased from Tainuo (Shanghai, China).

### Experimental animals

All procedures involving animals were approved by the Animal Ethics Committee of the Tianjin International Joint Academy of Biotechnology and Medicine. Eight-week old, pathogen-free female C57BL/6 mice and female Balb/C mice were purchased from the Animal Center Academy of Military Medical Science (Beijing, China) and acclimatized for 7 days before the experiment.

### Induction and treatment of chronic UC

The effects of apigenin on colitis were investigated using a DSS-induced chronic UC model. Fortymice were divided among the following groups according to average body weight: normal control group (Control), UC model control group (Model), and two apigenin dose groups (low- and high-dose apigenin groups treated with 200 and 300 mg/kg apigenin, respectively). Model and apigenin group mice received drinking water with 1% w/v DSS for 21 days based on published protocols. Apigenin was orally administered once daily from day 2 to day 21, while mice in the control groups received 0.5% CMC-Na intragastrically once per day. A schematic of the animal model established in this study and the apigenin administration methods is shown in Figure 1A.

### Treatment with apigenin

Inflammation and injury in the colons of model mice were observed after 2 days, and mice received apigenin solution (200 mg/kg (low dose) or 300 mg/kg (highdose)) via oral gavage once per day for 20 days. The control mice received drinking water intragastrically once per day. Body weight, stool consistency, and hematochezia data were recorded daily for the duration of the experiment. Colitis was evaluated based on the indicators mentioned above.

### CAC induction and treatment

After Balb/C mice (n = 40) reached target weights of 18-20 g, they were randomly assigned to one of the four experimental groups described above. For CAC studies, the mice received intraperitoneal injections of AOM (12 mg/kg) dissolved in physiological saline. After 5 days, the mice received drinking water with 2% w/v DSS for 7 days followed by a two-week recovery period, during which mice received DSS-free water. Each induction cycle lasted for 21 days; DSS treatment was repeated three times to establish the CAC animal model. Mice were sacrificed after 68 days. Mice in the control and model groups were treated with physiological saline instead of AOM and DSS. The treatment groups received either 200 or 300 mg/kg apigenin daily via oral gavage at the beginning of third cycle. On day 68, the mice were sacrificed and blood and colon samples were collected for further study. Colon length and tumor counts and size were measured. Tissues of the distal colon were then fixed in 4% paraformaldehyde for at least 24h, and histopathological and immunohistochemical analyses of paraffin-embedded sections were performed. The sections were stained with hematoxylin-eosin (H&E) in accordance with standard protocols, and immunohistochemistry (IHC) was used to determine levels of the colon cancer markers P53, CEA, CK8, and CK18. Disease activity index (DAI) scores were calculated based on stool consistency, fecal occult blood, and weight loss.

### Cell lines and culture

The HCT-116 human colon cancer cell line was purchased from Keygen Biotech (Nanjing, China). The cells were cultured in RPMI-1640 medium supplemented with penicillin (100 units/mL), streptomycin (100 mg/mL), and fetal bovine serum (10%) at 37 °C in a 5% CO_2_ atmosphere.

### Western blot analysis

Cell samples were scraped a few minutes after addition of lysis liquid, and protein concentrations in cell lysates were measured using a BCA kit. Total protein (30 μg per lane) was loaded onto a SDS-polyacrylamide gel. Bands were thentransferred to a polyvinylidenedifluoride membrane, blocked, and probed with antibodies (anti-p-NF-κB-p65, anti-T-NF-κB-p65, anti-p-state-3, anti-T-state-3, and anti-β-actin). Binding of antibody to antigen was detected using enhanced ECLprime (GE Healthcare) and captured and analyzed using a Las-3000luminescent Image Analyzer (Fuji Film).

### Dual-luciferase reporter assays

HCT-116 cells were plated in 96-well platesat a density of 6.0×10^4^ cells per well. NF-κB-luc and State-3-luc plasmids containing the response element were transfected into the cells; following replacement with fresh medium 24 h after transfection, the cells were treated with various concentrations of apigenin. Cell lysate (NF-κB and STAT-3) was collected on a96-well white plate after 48 h, and luciferase activity was detected using a luciferase reporter assay.

### ELISA

Colon tissues were homogenized with lysis buffer, centrifuged, and total protein was quantifiedusing a BCA proteinassay kit. Levels of myeloperoxidase (MPO) and other cytokineswere quantified using an ELISA kit according to the manufacturer’s instructions.

### H&E and IHC staining

Paraffin-embedded sections (4 μm) obtained from the colitis model group mice were stained with H&E according to standard protocols, and sections obtained from AOM/DSS-CAC model mice were stained with anti-P53, anti-CEA, anti-CK8, anti-CK18 antibodies. Sections were then incubated with biotin-labelled secondary antibody and streptavidin-HRP at room temperature. Immunoreactions were visualized using 3-3-diaminobenzidine and counterstained with hematoxylin. Histopathological analysis was then performed.

### Statistical analysis

All values are presented as means ± SD. Differences between groups were analyzed by Mann–Whitney U tests and ANOVAs when appropriate. Statistical analyses were performed using SPSS 17.0 (SPSS, Inc., Chicago, IL, USA). A *P* value of < 0.05 indicated statistical significance.

## Abbreviations

DSS: dextran sulfate sodium
LPS: lipopolysaccharide
IBD: inflammatory bowel disease
CAC: colitis-associated cancer
